# Medicaid Retention After Transition to Medicare Among Adults With Expansion Coverage

**DOI:** 10.1001/jamanetworkopen.2026.8560

**Published:** 2026-04-22

**Authors:** Zhiyou Yang, David Cheng, Mary Price, Kobi Khong, Timothy Duong, J. Wyatt Koma, John Hsu, Margarita Alegria, Joseph P. Newhouse, Vicki Fung

**Affiliations:** 1 The Mongan Institute, Massachusetts General Hospital, Boston; 2 Department of Medicine, Harvard Medical School, Boston, Massachusetts; 3 Biostatistics Center, Massachusetts General Hospital, Boston; 4 Interfaculty Initiative in Health Policy, Harvard University, Cambridge, Massachusetts; 5 Harvard Kennedy School, Cambridge, Massachusetts; 6 Department of Health Policy and Management, Harvard T.H. Chan School of Public Health, Boston, Massachusetts; 7 National Bureau of Economic Research, Cambridge, Massachusetts; 8 Department of Health Care Policy, Harvard Medical School, Boston, Massachusetts

## Abstract

**Importance:**

When becoming eligible for Medicare, adults previously covered by Medicaid expansion are typically required to meet an asset test for Medicaid coverage of Medicare cost-sharing and/or premiums through the Medicare Savings Program (MSP). It is unclear whether MSP or overall Medicaid coverage is higher in states that waive the MSP asset test than in states that do not.

**Objective:**

To examine patterns of Medicaid coverage in the first year of Medicare enrollment among beneficiaries who previously enrolled in Medicaid through expansion.

**Design, Setting, and Participants:**

This cohort study used Medicare and Medicaid enrollment data from January 1, 2017, to December 31, 2019, to identify beneficiaries who entered Medicare in 2018 and had Medicaid expansion coverage in the month prior to entering Medicare. The analysis included 31 states plus Washington, DC, that adopted Medicaid expansion by January 2018, of which 6 states (Arizona, Connecticut, Delaware, New York, Oregon, and Vermont) plus Washington, DC, waived the MSP asset test. Analyses were conducted between May 2024 and February 2026.

**Exposure:**

States without vs states with the MSP asset test.

**Main Outcomes and Measures:**

MSP or any Medicaid coverage 1 year after Medicare entry and at least 6 months or 12 months of MSP or any Medicaid coverage during the first year of Medicare enrollment. Multivariable linear probability models were used to examine the probability of these outcomes among beneficiaries living in states without vs states with the MSP asset test.

**Results:**

A total of 266 009 adults with Medicaid expansion coverage entered Medicare in 2018. These beneficiaries had a mean (SD) age of 59 (9) years and included 141 821 females (53%). Overall, 62% of beneficiaries entered Medicare due to reaching 65 years or older; 79% of beneficiaries lived in states with the MSP asset test and 21% lived in states without the MSP asset test. At the 12th month of their first year of Medicare enrollment, 48% had MSP coverage (48% vs 47% in states with vs without the MSP asset test, respectively), 61% had any Medicaid coverage (59% vs 67%, respectively). In adjusted analyses, beneficiaries in states without the MSP asset test were more likely than those living in states with the test to have any Medicaid coverage at the end of their first year of Medicare enrollment (difference, 10 [95% CI, 3-17] percentage points) and to have at least 6 months (difference, 14 [95% CI, 5-22] percentage points) or 12 months (difference, 16 [95% CI, 6-26] percentage points) of Medicaid coverage in their first year, but they were not more likely to have MSP coverage on these measures.

**Conclusions and Relevance:**

In this cohort study of adults with Medicaid expansion coverage entering Medicare in 2018, Medicaid coverage levels after Medicare entry were higher in states that waived the MSP asset test. The substantial share of enrollees without MSP or other Medicaid coverage, however, raises concerns about the incomplete uptake of available financial assistance among low-income Medicare beneficiaries.

## Key Points

**Question:**

Among low-income adults with Medicaid expansion coverage, how often do they retain Medicaid after transitioning to Medicare, and is retention higher in states without the Medicare Savings Program (MSP) asset test?

**Findings:**

In this cohort study of 266 009 adults with Medicaid expansion coverage who entered Medicare in 2018, 37% lacked Medicaid coverage by the end of their first year of Medicare enrollment. Medicaid, but not MSP, coverage was higher in states that waived the MSP asset test than states that did not.

**Meaning:**

The findings of this study suggest that waiving the MSP asset test could help beneficiaries retain Medicaid coverage when transitioning to Medicare, but many of them appear to miss opportunities to enroll in MSPs.

## Teaser

This cohort study examines the level of uptake of Medicare Savings Program or other Medicaid programs throughout the first year of Medicare enrollment among low-income beneficiaries in states with vs without the asset test.

## Introduction

To date, 40 US states and Washington, DC, have adopted the Patient Protection and Affordable Care Act’s Medicaid expansion eligibility pathway for nondisabled adults younger than 65 years with an income up to 138% of the federal poverty level (FPL) based on Modified Adjusted Gross Income (MAGI).^1,2^ More than 20 million adults were enrolled in Medicaid through the expansion pathway in December 2024.^3^ However, this pathway is unavailable to individuals once they enroll in Medicare.^4^ While Medicare beneficiaries may qualify for Medicaid through non–MAGI pathways for individuals 65 years or older or with disabilities, the criteria are often more stringent than the expansion pathway criteria, with similar or lower income limits and an additional asset test.^5^ Moreover, because Medicare beneficiaries must undergo a new Medicaid eligibility determination after qualifying for Medicare, the administrative burdens of this process may discourage or delay Medicaid enrollment even among those eligible.^6,7^ There are limited data on the extent to which adults with expansion coverage qualify for Medicaid after they transition to Medicare.

Medicare beneficiaries with low income and limited assets can qualify for partial Medicaid benefits through the Medicare Savings Program (MSP), which covers Medicare cost-sharing and/or premiums.^8^ The upper-income limit of MSPs, typically 135% of the FPL, is similar to that of Medicaid expansion, and MSPs use more generous income-counting rules.^9^ In most states, however, beneficiaries must meet an asset test to be eligible for MSP coverage; in 2026, assets were limited to $9950 for an individual and $14 910 for a couple.^10^ The largest MSP eligibility group, Qualified Medicare Beneficiary, receives coverage for both Medicare Parts A and B cost-sharing and premiums.^8^ By March 2025, 17 states plus Washington, DC, had expanded eligibility for MSP coverage by increasing the income limits, increasing or waiving the asset limit, or both; waiving the asset test was the most common approach (used in 14 states plus Washington, DC).^5^

Medicare beneficiaries can also qualify for full Medicaid benefits through other Medicaid eligibility pathways for older adults and people with disabilities, most commonly through Supplemental Security Income (SSI)-related pathways, which are generally available to individuals with an income up to 74% of the FPL and assets up to $2000.^11^ By March 2025, 28 states plus Washington, DC, had opted to increase the income limit, most commonly to 100% of the FPL, and/or increase the asset limit.^5^ Beneficiaries with full-benefit Medicaid may or may not be enrolled in MSPs.^12,13,14^ Either way, Medicaid typically pays Medicare premiums and cost-sharing for individuals with full benefit dual-eligibility status.^15^ In addition, all beneficiaries with full and partial dual-eligibility status automatically qualify for Medicare Part D’s Low-Income Subsidy (LIS), which helps pay premiums and most cost-sharing for prescription drug coverage.^16,17^ Additional details on Medicaid eligibility pathways for Medicare beneficiaries are available in eTable 1 in Supplement 1.

In contrast to Medicaid, which is required to maintain low to no cost-sharing and premiums, Medicare cost-sharing and premiums can be substantial. For example, in 2026, Medicare Part B has a base premium of $203 per month, a deductible of $283, and 20% coinsurance.^18^ Thus, the transition from Medicaid to Medicare may result in substantial increases in out-of-pocket costs if beneficiaries do not qualify for or enroll in Medicaid after entering Medicare. Waiving the MSP asset test could reduce eligibility and administrative barriers to enrolling in Medicaid after Medicare entry, but there is limited information on the association between state MSP eligibility policies and Medicaid enrollment.

We therefore aimed to examine patterns of Medicaid coverage in the first year of Medicare enrollment among beneficiaries who were previously enrolled in Medicaid through expansion. We assessed whether these beneficiaries were more likely to have MSP or any Medicaid coverage in states that waived (without) vs states that did not waive (with) the MSP asset test.

## Methods

### Data Sources and Study Sample

We used linked Transformed Medicaid Statistical Information System Analytic Files (TAF) and the Medicare Beneficiary Summary File (MBSF) from January 1, 2017, to December 31, 2019. We focused on this period to avoid overlap with the start of the Medicaid continuous enrollment provision that was implemented in March 2020.^19^ We used the MBSF to identify beneficiaries who entered Medicare in 2018, and we used the TAF demographic and eligibility file to identify those with Medicaid expansion coverage in the month prior to entering Medicare. Our analysis included 31 states plus Washington, DC, that adopted Medicaid expansion by January 2018, among which 6 states (Arizona, Connecticut, Delaware, New York, Oregon, and Vermont) plus Washington, DC, waived the MSP asset test in 2018. We excluded beneficiaries who changed states during their observation period. The Mass General Brigham Institutional Review Board deemed this study exempt from ethics review and informed consent requirement because the project met the criteria for secondary research. We followed the Strengthening the Reporting of Observational Studies in Epidemiology (STROBE) reporting guideline.

### Medicaid Coverage

We assessed whether beneficiaries had any Medicaid coverage in each of the first 12 months of Medicare enrollment. We classified beneficiaries as enrolled in MSPs (with or without full-benefit Medicaid) or in other full-benefit Medicaid programs without MSPs, using the monthly dual-eligibility codes in the MBSF. For beneficiaries without Medicaid coverage, we examined whether they had Medicare Part D LIS coverage. The full Part D LIS had the same income and asset limits as the federal MSP limits in this period.^20^ Although the income- and asset-counting rules differ slightly among states, beneficiaries without Medicaid but with Part D LIS coverage were likely to be eligible for MSPs but unenrolled.^20^ Furthermore, we determined whether beneficiaries had missing data on Medicaid or Part D LIS status or died in each month.

### Statistical Analysis

We examined state-level percentages of beneficiaries who had MSP (with or without full-benefit Medicaid) or any Medicaid (ie, MSP or full-benefit Medicaid without MSP) coverage in their 12th month of Medicare enrollment using box and whisker plots stratified by whether states had the MSP asset test. We also examined overall percentages of beneficiaries with MSP or any Medicaid coverage in their 12th month of Medicare enrollment or for at least 6 or 12 months during their first year of Medicare enrollment.

We fit multivariable linear probability models to examine differences in the probability of these Medicaid coverage outcomes for beneficiaries in states without vs states with the MSP asset test. We adjusted for several covariates: beneficiary’s reason for Medicare entitlement (age, disability, or end-stage kidney disease), sex, race and ethnicity (using Research Triangle Institute race codes: American Indian or Alaska Native, Asian or Pacific Islander, Black or African American, Hispanic, non-Hispanic White, Other, and Unknown; we combined American Indian or Alaska Native and Unknown with Other due to small cell sizes), and residency in a metropolitan county (as defined by the United States Department of Agriculture Rural-Urban Continuum Codes^21^ 1-3). Race and ethnicity data were obtained from the MBSF and included in our analysis to control for potential confounding.

Using a 12-month lookback period before the month of each beneficiary’s Medicare entry, we adjusted for whether the beneficiary had 12 months of continuous Medicaid enrollment, any Medicaid comprehensive managed care organization (MCO) enrollment, and any use of Medicaid inpatient care or long-term services and supports. We also adjusted for the Centers for Medicare & Medicaid Services Hierarchical Condition Category (CMS-HCC) score using diagnoses from Medicaid claims during the 1-year lookback period^22^ and for beneficiaries’ neighborhood socioeconomic status using the 2018 Centers for Disease Control and Prevention and Agency for Toxic Substances and Disease Registry Social Vulnerability Index (CDC/ATSDR SVI) based on 5-digit zip codes^23^; we operationalized the CMS-HCC and CDC/ATSDR SVI scores as quartiles. In addition, we adjusted for other state Medicaid policies that could affect the probability of qualifying for full-benefit Medicaid, including whether states automatically enrolled individuals with SSI into Medicaid and whether states had optional poverty-related pathways (ie, an increased income limit of 100% of the FPL) or a medically needy pathway for older adults or people with disabilities.^24,25^ We clustered SEs at the state level.

Pearson χ^2^ tests were used to compare the covariates between the beneficiaries in states with vs states without the asset test, and *P* < .05 indicated statistical significance. Data analyses were conducted between May 2024 and February 2026 using Stata, versions 18 and 19 (StataCorp LLC).

In sensitivity analyses, we examined the differences in the percentage of beneficiaries with MSP coverage but without full-benefit Medicaid; such beneficiaries could be more sensitive to the elimination of the MSP asset test. We separately examined beneficiaries who qualified for Medicare due to age (≥65 years) vs due to disability. To address TAF data quality concerns, we conducted sensitivity analyses that excluded 5 states for which any of the following data elements were classified as unusable based on the CMS Data Quality (DQ) Atlas^26^: eligibility group code, expansion enrollment, and Medicaid comprehensive MCO enrollment in any year between 2017 and 2019.

## Results

### Characteristics of the Study Population

We identified 266 009 adults with Medicaid expansion coverage who transitioned to Medicare in 2018. Among them, 79% lived in states with the MSP asset test and 21% lived in states without, and 62% entered Medicare due to age. The sample had a mean (SD) age of 59 (9) years and included 141 821 females (53%) and 124 188 males (47%). Their race and ethnicity were categorized as follows: 9% as Asian or Pacific Islander, 14% as Black or African American, 19% as Hispanic, and 53% as non-Hispanic White; 4%, including American Indian or Alaska Native individuals, were grouped into the other category because of small numbers (Table 1).

**Table 1.** Characteristics of Beneficiaries With Expansion Coverage Transitioning to Medicare in 2018 in States With vs States Without the MSP Asset Test

Characteristic	Beneficiaries, No. (%)			*P* value^a^
	All (N = 266 009)	In states with MSP asset test (n = 211 374)	In states without MSP asset test (n = 54 635)	
Original reason for Medicare entitlement				
Age	164 059 (62)	127 723 (60)	36 336 (67)	<.001
Disability	98 504 (37)	80 879 (38)	17 625 (32)	
ESKD or both disability and ESKD	3446 (1)	2772 (1)	674 (1)	
Sex				
Female	141 821 (53)	112 486 (53)	29 335 (54)	.047
Male	124 188 (47)	98 888 (47)	25 300 (46)	
Race and ethnicity				
Asian or Pacific Islander	24 628 (9)	18 083 (9)	6545 (12)	<.001
Black or African American	37 424 (14)	30 011 (14)	7413 (14)	
Hispanic	50 691 (19)	39 373 (19)	11 318 (21)	
Non-Hispanic White	141 724 (53)	115 538 (55)	26 186 (48)	
Other^b^	11 542 (4)	8369 (4)	3173 (6)	
CMS-HCC score quartile				
1, Most healthy	77 804 (29)	61 656 (29)	16 148 (30)	<.001
2	55 668 (21)	44 032 (21)	11 636 (21)	
3	66 092 (25)	52 450 (25)	13 642 (25)	
4, Least healthy	66 445 (25)	53 236 (25)	13 209 (24)	
1 y Before Medicare entry				
12-mo Continuous Medicaid enrollment	230 755 (87)	184 275 (87)	46 480 (85)	<.001
Any Medicaid comprehensive MCO enrollment	219 814 (83)	173 563 (82)	46 251 (85)	<.001
Any Medicaid inpatient utilization	34 692 (13)	27 596 (13)	7096 (13)	.68
Any Medicaid LTSS utilization	5310 (2)	4316 (2)	994 (2)	.001
Live in a metropolitan county				
Yes	224 662 (84)	175 713 (83)	48 949 (90)	<.001
No	39 808 (15)	34 382 (16)	5426 (10)	
Missing data	1539 (1)	1279 (1)	260 (0)	
CDC/ATSDR SVI quartile				
1, Least vulnerable	66 448 (25)	53 789 (25)	12 659 (23)	<.001
2	66 569 (25)	53 914 (26)	12 655 (23)	
3	63 509 (24)	48 657 (23)	14 852 (27)	
4, Most vulnerable	64 911 (24)	51 169 (24)	13 742 (25)	
Missing data	4572 (2)	3845 (2)	727 (1)	
State SSI-related Medicaid enrollment; optional poverty-related pathway for older adults and people with disabilities				
Without autoenrollment; without increased income limit of 100% FPL	15 774 (6)	6287 (3)	9487 (17)	<.001
Without autoenrollment; with increased income limit of 100% FPL	25 867 (10)	25 867 (12)	0	
With autoenrollment; without increased income limit of 100% FPL	109 567 (41)	72 210 (34)	37 357 (68)	
With autoenrollment; with increased income limit of 100% FPL	114 801 (43)	107 010 (51)	7791 (14)	
State optional medically needy Medicaid pathway	215 576 (81)	173 765 (82)	41 811 (77)	<.001

Abbreviations: CDC/ATSDR SVI, Centers for Disease Control and Prevention and Agency for Toxic Substances and Disease Registry Social Vulnerability Index; CMS-HCC, Centers for Medicare & Medicaid Services Hierarchical Condition Category; ESKD, end-stage kidney disease; FPL, federal poverty level; LTSS, long-term services and supports; MCO, managed care organization; MSP, Medicare Savings Program; SSI, Supplemental Security Income.

^a^ *P* values calculated using Pearson χ^2^ tests.

^b^ Other race and ethnicity included American Indian or Alaska Native and unknown.

Overall, beneficiaries in states without compared with states with the MSP asset test were more likely to reach the eligibility age of Medicare or to reside in a metropolitan county and were less likely to be non-Hispanic White (Table 1). States without the MSP asset test were also more likely to lack optional poverty-related or medically needy Medicaid pathway for older adults and people with disabilities.

### Medicaid Coverage in the First Year of Medicare Enrollment

**Figure 1.**
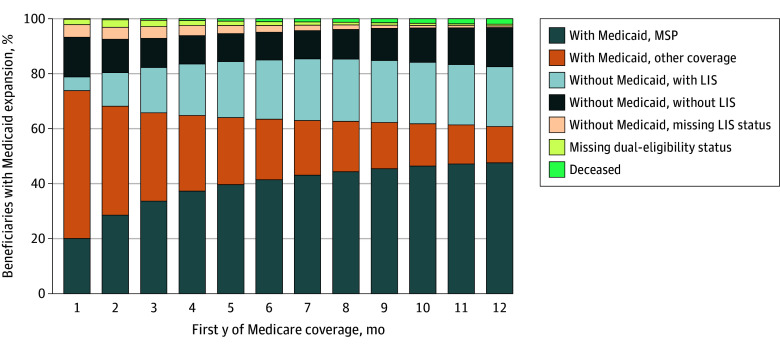
Component Bar Graph of Monthly Medicaid Coverage in the First Year of Medicare Transition (2018) Among Beneficiaries With Expansion Coverage LIS indicates low-income subsidy, and MSP indicates Medicare Savings Program.

Among adults with expansion coverage entering Medicare, the percentage with MSP coverage increased from 20% in their first month to 48% in their 12th month of Medicare enrollment. By the 12th month, 61% had any Medicaid coverage, 22% had Medicare Part D LIS but not Medicaid coverage, and 37% lacked Medicaid coverage (Figure 1).

### State-Level Variation in Medicaid Coverage

**Figure 2.**
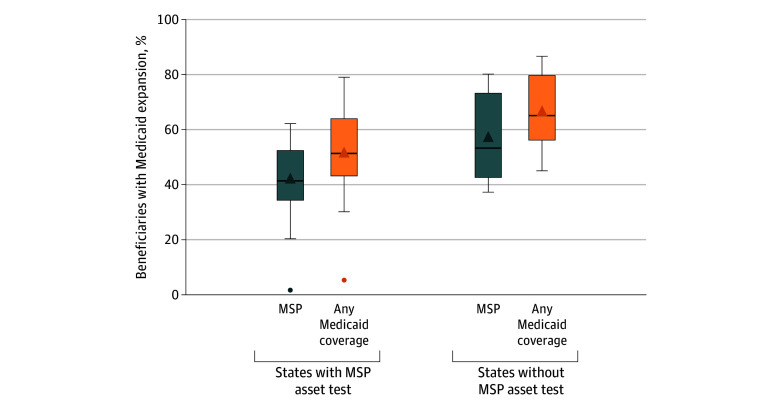
Box and Whisker Plot of Medicaid Coverage in the First Year of Medicare Transition (2018) Among Beneficiaries With Expansion Coverage in States With vs States Without the Medicare Savings Program (MSP) Asset Test Upper and lower ends of the boxes represent the third and first quartile, respectively; horizontal line inside boxes represent the median; whiskers represent the minimum and maximum values (excluding outliers). Triangles represent the mean, and circles below or above boxes represent outliers, defined as data points that lie more than 1.5 times the IQR above the third quartile or below the first quartile.

There was wide variation in the state-level percentage of beneficiaries with MSP or any Medicaid coverage 1 year after Medicare entry (eFigure 1 in Supplement 1). The median state-level percentage of beneficiaries with MSP was higher in states without than states with the MSP asset test (53% vs 41%), and so was the interstate variation as measured by the IQR (73%-43% vs 52%-34%) (Figure 2). Patterns were similar for beneficiaries with any Medicaid coverage (median: 65% vs 51%; IQR: 80%-56% vs 64%-43%).

### Medicaid Coverage in States Without vs States With the MSP Asset Test

The overall percentage of beneficiaries with MSP coverage in their 12th month of Medicare enrollment was similar in states without vs states with the MSP asset test (47% vs 48%) (eFigure 2 in Supplement 1). However, the percentage of beneficiaries with any Medicaid coverage was higher in states without vs states with the MSP asset test (67% vs 59%).

The percentage of beneficiaries with at least 6 or 12 months of MSP or any Medicaid coverage followed similar patterns. For example, in states without vs states with the MSP asset test, 41% vs 45% had at least 6 months of MSP coverage and 75% vs 64% had at least 6 months of any Medicaid coverage, excluding beneficiaries who had missing dual-eligibility status data or were deceased (Table 2).

**Table 2.** Associations Between the MSP Asset Test and Medicaid Coverage Over the First Year of Medicare Enrollment Among Beneficiaries With Expansion Coverage^a^

Outcome	Beneficiaries, No. (%)		Adjusted difference between states with vs without MSP asset test (95% CI), pp
	In states with MSP asset test	In states without MSP asset test	
**MSP coverage**			
In the 12th mo after Medicare entry	101 046 (49)	25 464 (48)	−1 (−12 to 10)
At least 6 mo during the first Medicare y	89 177 (45)	20 699 (41)	−2 (−17 to 12)
12 mo during the first Medicare y	34 117 (17)	6849 (14)	−1 (−19 to 17)
**Any Medicaid coverage** ^b^			
In the 12th mo after Medicare entry	124 857 (61)	36 666 (69)	10 (3 to 17)
At least 6 mo during the first Medicare y	127 784 (64)	37 654 (75)	14 (5 to 22)
12 mo during the first Medicare y	92 825 (47)	29 646 (59)	16 (6 to 26)

Abbreviations: MSP, Medicare Savings Program; pp, percentage point.

^a^ Both unadjusted and adjusted results excluded beneficiaries with missing dual-eligibility status or deceased in the relevant month of the outcome (Figure 1). Adjusted models excluded beneficiaries with American Indian or Alaska Native, Other, or Unknown race and ethnicity or with missing United States Department of Agriculture metropolitan county data or Centers for Disease Control and Prevention Social Vulnerability Index quartile data (Table 1). SEs were clustered at the state level.

^b^ Any Medicaid included MSP coverage (with or without full-benefit Medicaid) and full-benefit Medicaid without MSP coverage.

The adjusted and unadjusted differences in Medicaid coverage outcomes for beneficiaries in states without vs states with the MSP asset test were consistent. There were no significant differences in MSP enrollment outcomes between beneficiaries in states without vs states with the MSP asset test. However, beneficiaries in states without the MSP asset test were more likely to have any Medicaid coverage in their 12th month of Medicare enrollment (difference, 10 [95% CI, 3-17] percentage points [pp]) and more likely to have at least 6 months (difference, 14 [95% CI, 5-22] pp) or 12 continuous months (difference, 16 [95% CI, 6-26] pp) of any Medicaid coverage during their first year of Medicare enrollment (Table 2; eTables 2 and 3 in Supplement 1).

### MSP Without Full-Benefit Medicaid Coverage

The percentage of beneficiaries with MSP but without full-benefit Medicaid coverage was similar in states without vs states with the MSP asset test (eFigure 3 and eTable 4 in Supplement 1). In analyses stratified by reason for Medicare entitlement, beneficiaries who qualified due to disability rather than due to age were less likely to be enrolled in MSPs (42% vs 51%) and more likely to have full-benefit dual-eligibility status without MSP coverage (19% vs 9%) by the end of their first year of Medicare enrollment (eFigure 4 in Supplement 1); however, associations between the MSP asset test and Medicaid coverage outcomes were similar to associations observed in the main analyses (eTable 5 in Supplement 1). In analyses excluding 5 states with TAF data quality concerns, the findings were also consistent (eTables 2 and 3 in Supplement 1).

## Discussion

We found that among adults with Medicaid expansion coverage who transitioned to Medicare in 2018, only 48% were enrolled in MSPs and only 61% had any Medicaid coverage by the end of their first year of Medicare enrollment. On average, there was greater continuous Medicaid coverage after Medicare entry in states without vs states with the MSP asset test, even after controlling for differences in eligibility criteria for other Medicaid pathways for older adults and people with disabilities. The probability of having MSP coverage, however, did not differ between beneficiaries in states without vs states with the MSP asset test during their first year of Medicare enrollment.

The limited association between state waivers of the MSP asset test and MSP enrollment was unexpected given previous reports from Medicaid administrators and beneficiaries that identified the asset test as a considerable source of administrative burden and a barrier to enrollment.^27^ When comparing state-level MSP coverage, the median percentage was higher in states without vs states with the asset test. Still, there was wide variation across states, particularly among states without the asset test. State variability in MSP enrollment highlights the importance of state outreach and other enrollment processes in facilitating Medicaid and MSP enrollment. The MACPAC (Medicaid and Children's Health Insurance Program Payment and Access Commission) has previously recommended that states increase funding for MSP education and outreach, clarify notices and information about MSP for individuals covered by Medicaid who are transitioning to Medicare, and adopt streamlined enrollment processes such as prepopulated enrollment forms.^28^ Tracking state outreach or enrollment processes for non-MAGI eligibility groups is critical for assessing their implication for coverage.

Fifty-one percent of adults with Medicaid expansion coverage in states without the MSP asset test did not have MSP at the end of their first year of Medicare enrollment. This finding raises concerns about the incomplete uptake of available Medicaid assistance among eligible beneficiaries because the income limits for Medicaid expansion and MSPs are similar (ie, 138% vs 135% of the FPL). MSPs use more generous income-counting rules, and the federal MSP asset test did not apply in these states. Medicaid and Medicare enrollment data do not include information on individual-level income; thus, anchoring on the Medicaid expansion population strengthens our ability to observe MSP enrollment among low-income individuals who are likely to be eligible, since most beneficiaries who qualify for MSPs based on income have limited assets.^29^ Our findings are consistent with estimates from survey data reporting that only about half to two-thirds of eligible beneficiaries enroll in MSPs.^9,30,31^ Additionally, across all states, we found that 22% of beneficiaries who transitioned from Medicaid expansion to Medicare coverage were enrolled in Part D LIS without Medicaid by the end of their first year of Medicare enrollment. Given that the income and asset limits for full-benefit Part D LIS were nearly identical to limits for the MSP Qualifying Individuals group, this finding suggests a sizable number of eligible but unenrolled beneficiaries.

These findings underscore the administrative difficulties beneficiaries may face in navigating Medicare entry, which involves numerous, interdependent coverage decisions and new Medicaid eligibility pathways and rules.^6^ Decisions to enroll in Medicare Advantage, for example, may be affected by whether beneficiaries receive MSP coverage for Medicare premiums and cost-sharing, as are Medigap and Part D coverage choices among those in traditional Medicare. Qualitative work has found that adults with expansion coverage find the process of enrolling in Medicare to be complex and the eligibility rules for dual eligibility to be confusing,^7^ highlighting the need to simplify these processes and provide additional enrollment assistance to beneficiaries.

To streamline Medicaid enrollment, including in MSPs, CMS issued 2 final rules in 2023 and 2024, collectively referred to as the Eligibility and Enrollment Final Rule. These rules codified requirements for states to use information from the Social Security Administration on Part D LIS applications as the MSP applications by April 1, 2026.^12,14^ Additionally, they allowed some states to automatically enroll beneficiaries with SSI into the Qualified Medicare Beneficiary eligibility group by October 2024^12,14^ and required states to align the application and renewal processes for MAGI and non–MAGI eligibility groups by June 2027.^32,33^

The Budget Reconciliation Act of 2025, however, delayed implementation of these rules until October 1, 2034.^34^ The Congressional Budget Office estimated that repealing these rules would decrease the number of Medicare enrollees with Medicaid by approximately 1.4 million people over 10 years.^35^ In addition, substantial losses in overall Medicaid expansion enrollment resulting from the Budget Reconciliation Act of 2025 are likely to further depress Medicaid participation among eligible Medicare beneficiaries because states will have fewer touchpoints to identify and assist low-income individuals transitioning to Medicare. Assessing how these policies affect Medicaid enrollment, utilization, and outcomes among low-income Medicare beneficiaries is crucial, as reducing the uptake of available financial assistance will likely exacerbate the persistent health care affordability challenges faced by this population.^30,36,37^

### Limitations

This study has limitations. First, we could not assess causal associations between the MSP asset test and Medicaid enrollment. Second, we were unable to determine whether beneficiaries who were not enrolled in Medicaid were ineligible or were eligible but unenrolled. Enrollment is capped each year for 1 MSP eligibility group (Qualifying Individuals), with incomes between 120% and 135% of the FPL,^10,38^ but we could not identify if states reached their cap. Lastly, data were not yet available on enrollment patterns after the Medicaid continuous enrollment provision ended in April 2023^19^; therefore, our results may not reflect the most current circumstances. Since 2018, 6 more states (California, Louisiana, Maine, Massachusetts, New Mexico, and Washington) have waived the MSP asset test.^5,25^ California also expanded full-benefit Medicaid eligibility in 2020 to older adults and people with disabilities with incomes at or below 138% of the FPL^39^ and waived the asset test in 2024 but reinstated it in 2026.^25,40^

## Conclusions

This cohort study of adults with Medicaid expansion coverage entering Medicare in 2018 showed that 48% of these beneficiaries had MSP coverage and 61% had any Medicaid coverage by the end of their first year of Medicare enrollment. Beneficiaries in states that waived the MSP asset test were more likely to have any Medicaid coverage after entering Medicare compared with those in states with the asset test. However, persistent gaps in MSP enrollment and variability across states underscore the need for policies and processes that facilitate a smooth transition from Medicaid to Medicare for low-income adults. These interventions would mitigate the affordability challenges associated with Medicare premiums and cost-sharing.

## Notes

Supplement 1. **eTable 1.** Medicaid Eligibility Pathways and Criteria for Medicare Beneficiaries **eFigure 1.** Medicaid Coverage One Year After Medicare Entry of Expansion Adults Transitioning to Medicare in 2018, by State **eFigure 2.** Medicaid Coverage by Month Over the First Medicare Year of Expansion Adults Transitioning to Medicare in 2018, in States With Versus Without the MSP Asset Test **eFigure 3.** MSP Coverage Only Without Full Medicaid by Month Over the First Medicare Year of Expansion Adults Transitioning to Medicare in 2018, in States With Versus Without the MSP Asset Test **eFigure 4.** Medicaid Coverage by Month Over the First Medicare Year of Expansion Adults Transitioning to Medicare in 2018, in States With Versus Without the MSP Asset Test, by Reason for Medicare Eligibility **eTable 2.** Associations Between the MSP Asset Test and MSP Coverage Over the First Medicare Year of Expansion Adults Transitioning to Medicare in 2018, Full Results **eTable 3.** Associations Between the MSP Asset Test and Any Medicaid Coverage Over the First Medicare Year of Expansion Adults Transitioning to Medicare in 2018, Full Results **eTable 4.** Associations Between the MSP Asset Test and MSP Coverage Only Without Full Medicaid Over the First Medicare Year of Expansion Adults Transitioning to Medicare in 2018 **eTable 5.** Associations Between the MSP Asset Test and MSP or any Medicaid coverage Over the First Medicare Year of Expansion Adults Transitioning to Medicare in 2018, by Reason for Medicare Eligibility

Supplement 2.
Data Sharing Statement
