# Supplementary material for: Medicaid Retention After Transition to Medicare Among Adults With Expansion Coverage
Source: JAMA Netw Open. 2026 Apr 22;9(4):e268560. doi: 10.1001/jamanetworkopen.2026.8560 (PMC13103809; doi:10.1001/jamanetworkopen.2026.8560)
Supplement: Supplement 1. — eTable 1. Medicaid Eligibility Pathways and Criteria for Medicare Beneficiaries eFigure 1. Medicaid Coverage One Year After Medicare Entry of Expansion Adults Transitioning to Medicare in 2018, by State eFigure 2. Medicaid Coverage by Month Over the First Medicare Year of Expansion Adults Transitioning to Medicare in 2018, in States With Versus Without the MSP Asset Test eFigure 3. MSP Coverage Only Without Full Medicaid by Month Over the First Medicare Year of Expansion Adults Transitioning to Medicare in 2018, in States With Versus Without the MSP Asset Test eFigure 4. Medicaid Coverage by Month Over the First Medicare Year of Expansion Adults Transitioning to Medicare in 2018, in States With Versus Without the MSP Asset Test, by Reason for Medicare Eligibility eTable 2. Associations Between the MSP Asset Test and MSP Coverage Over the First Medicare Year of Expansion Adults Transitioning to Medicare in 2018, Full Results eTable 3. Associations Between the MSP Asset Test and Any Medicaid Coverage Over the First Medicare Year of Expansion Adults Transitioning to Medicare in 2018, Full Results eTable 4. Associations Between the MSP Asset Test and MSP Coverage Only Without Full Medicaid Over the First Medicare Year of Expansion Adults Transitioning to Medicare in 2018 eTable 5. Associations Between the MSP Asset Test and MSP or any Medicaid coverage Over the First Medicare Year of Expansion Adults Transitioning to Medicare in 2018, by Reason for Medicare Eligibility [file jamanetwopen-e268560-s001.pdf]

Supplemental Online Content

Yang Z, Cheng D, Price M, et al. Medicaid retention after transition to Medicare among adults with expansion coverage. *JAMA Netw Open*. 2026;9(4):e268560. doi:10.1001/jamanetworkopen.2026.8560

eTable 1. Medicaid Eligibility Pathways and Criteria for Medicare Beneficiaries
eFigure 1. Medicaid Coverage One Year After Medicare Entry of Expansion Adults Transitioning to Medicare in 2018, by State
eFigure 2. Medicaid Coverage by Month Over the First Medicare Year of Expansion Adults Transitioning to Medicare in 2018, in States With Versus Without the MSP Asset Test
eFigure 3. MSP Coverage Only Without Full Medicaid by Month Over the First Medicare Year of Expansion Adults Transitioning to Medicare in 2018, in States With Versus Without the MSP Asset Test
eFigure 4. Medicaid Coverage by Month Over the First Medicare Year of Expansion Adults Transitioning to Medicare in 2018, in States With Versus Without the MSP Asset Test, by Reason for Medicare Eligibility
eTable 2. Associations Between the MSP Asset Test and MSP Coverage Over the First Medicare Year of Expansion Adults Transitioning to Medicare in 2018, Full Results
eTable 3. Associations Between the MSP Asset Test and Any Medicaid Coverage Over the First Medicare Year of Expansion Adults Transitioning to Medicare in 2018, Full Results
eTable 4. Associations Between the MSP Asset Test and MSP Coverage Only Without Full Medicaid Over the First Medicare Year of Expansion Adults Transitioning to Medicare in 2018
eTable 5. Associations Between the MSP Asset Test and MSP or any Medicaid coverage Over the First Medicare Year of Expansion Adults Transitioning to Medicare in 2018, by Reason for Medicare Eligibility

This supplemental material has been provided by the authors to give readers additional information about their work.

© 2026 Yang Z et al. *JAMA Network Open*

eTable 1. Medicaid eligibility pathways and criteria for Medicare beneficiaries

Program	Benefits	2018 Asset Limit		2018 Income Limit
		Individual	Couple	
A) Medicare Savings Programs (MSPs) eligibility groups				
Qualified Medicare Beneficiary (QMB): QMB Only or QMB Plus[(1)]	Part A and B premiums, cost-sharing	$7,560	$11,340	100% FPL
Specified Low-Income Medicare Beneficiary (SLMB): SLMB Only or SLMB Plus[(1)]	Part B premiums	$7,560	$11,340	120% FPL
Qualified Individual (QI)	Part B premiums (limited funding)	$7,560	$11,340	135% FPL
Qualified Disabled Working Individual (QDWI)[(2)]	Part A premiums	$4,000	$6,000	200% FPL
B) Eligibility pathways for full Medicaid benefits				
Supplemental Security Income (SSI) (mandatory)[(3)]	Full Medicaid benefits	$2,000	$3,000	74% FPL
Poverty Level Related (optional)	Full Medicaid benefits	$2,000	$3,000	Varies by state, commonly 100% FPL
Other optional Medicaid pathways (Medically Needy, Buy-in for Working People with Disabilities, Special Income Rule for Long-Term Services and Supports Eligibility, etc.)	Full Medicaid benefits	Varies by state and eligibility group and may exceed MSP limits		

Sources: Kaiser Family Foundation and CMS
Notes: (1) QMB Plus and SLMB Plus qualify for QMB or SLMB benefits in section A, as well as full Medicaid benefits through one of the eligibility pathways listed in section B. (2) QDWI has very limited enrollment; the majority of MSP enrollment is in QMB, SLMB, and QI. (3) Some states use different eligibility criteria.

eFigure 1. Medicaid coverage one year after Medicare entry of expansion adults transitioning to Medicare in 2018, by state

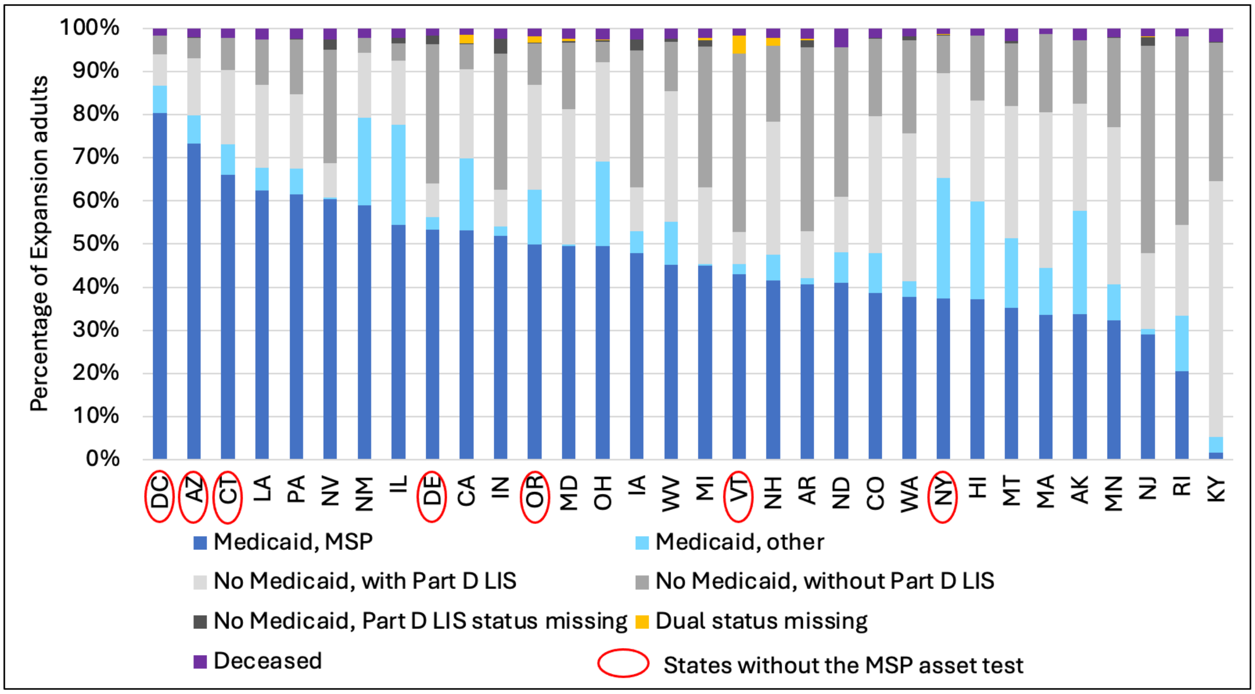

© 2026 Yang Z et al. *JAMA Network Open*

eFigure 2. Medicaid coverage by month over the first Medicare year of expansion adults transitioning to Medicare in 2018, in states with versus without the MSP asset test

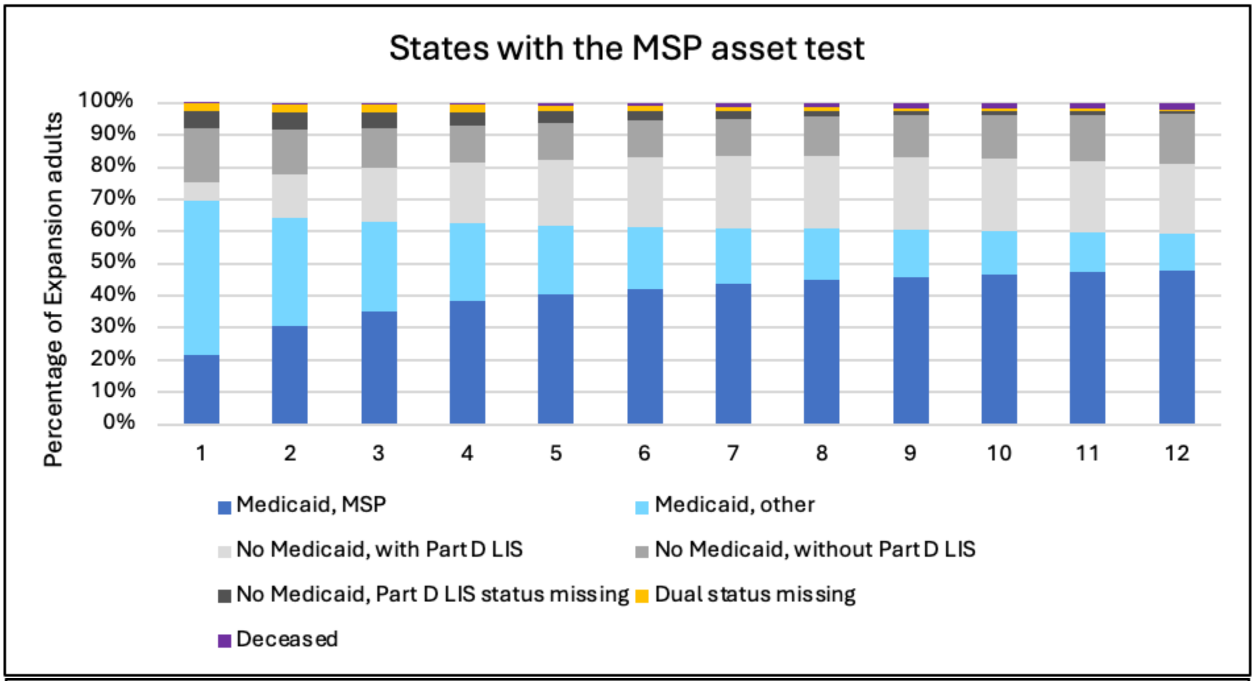

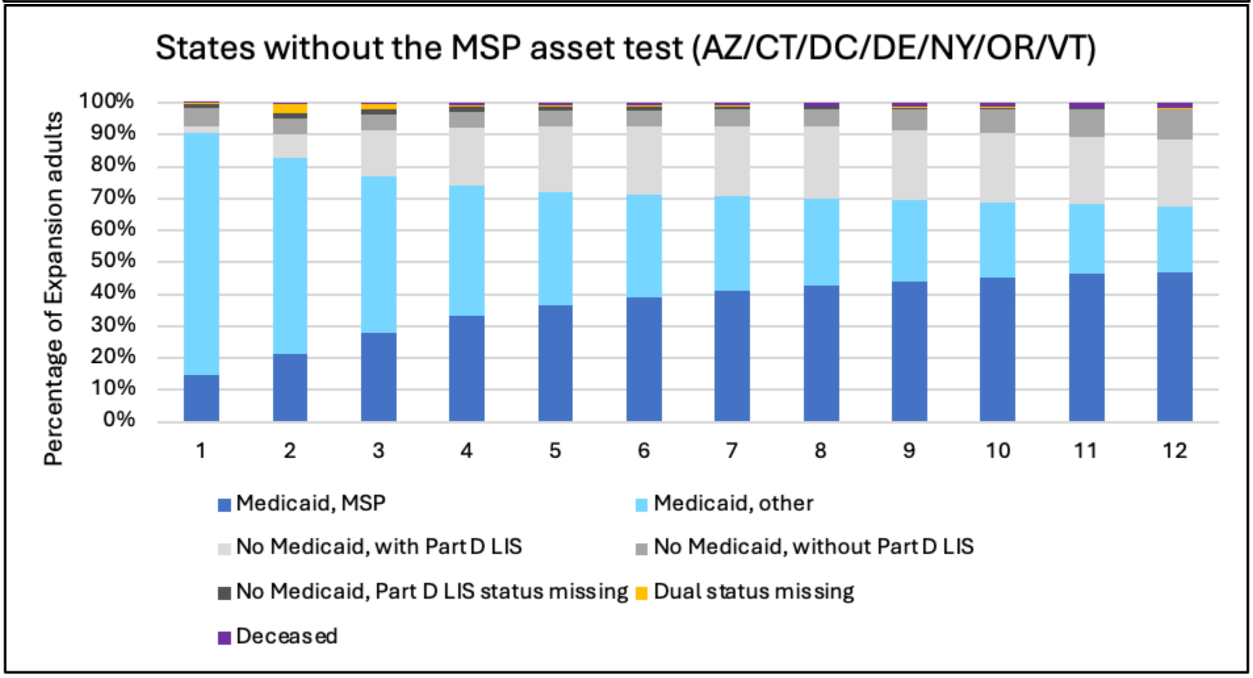

© 2026 Yang Z et al. *JAMA Network Open*

eFigure 3. MSP coverage only without full Medicaid by month over the first Medicare year of expansion adults transitioning to Medicare in 2018, in states with versus without the MSP asset test

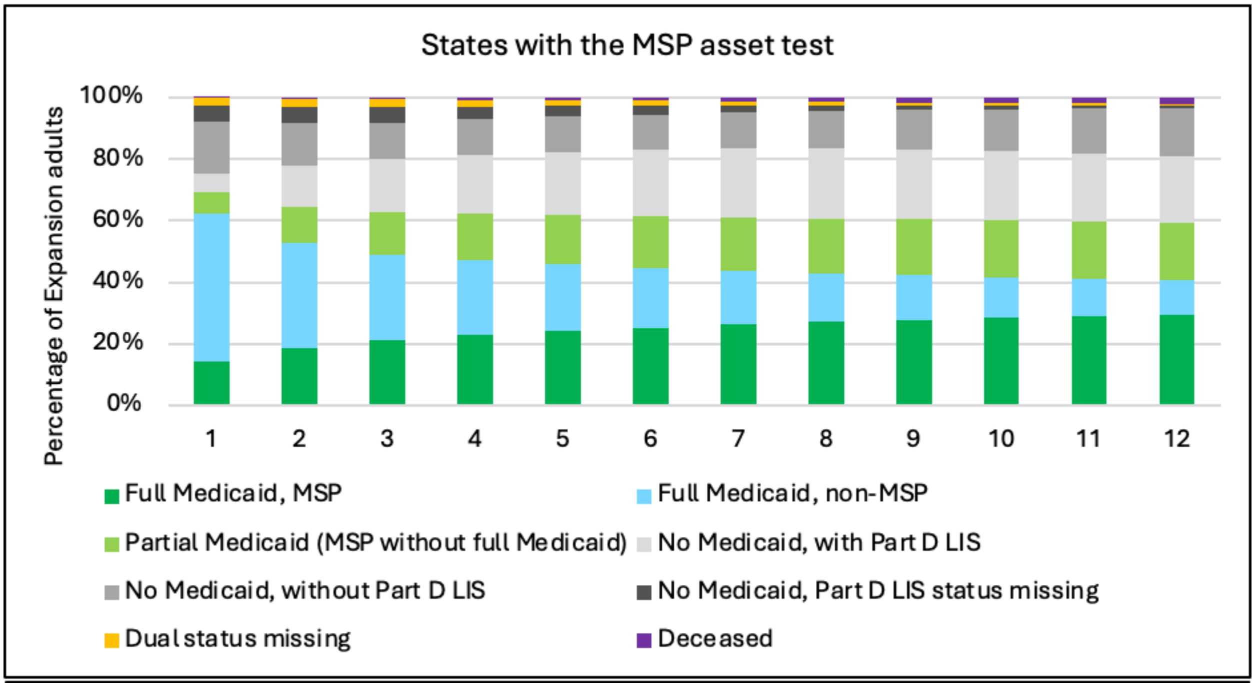

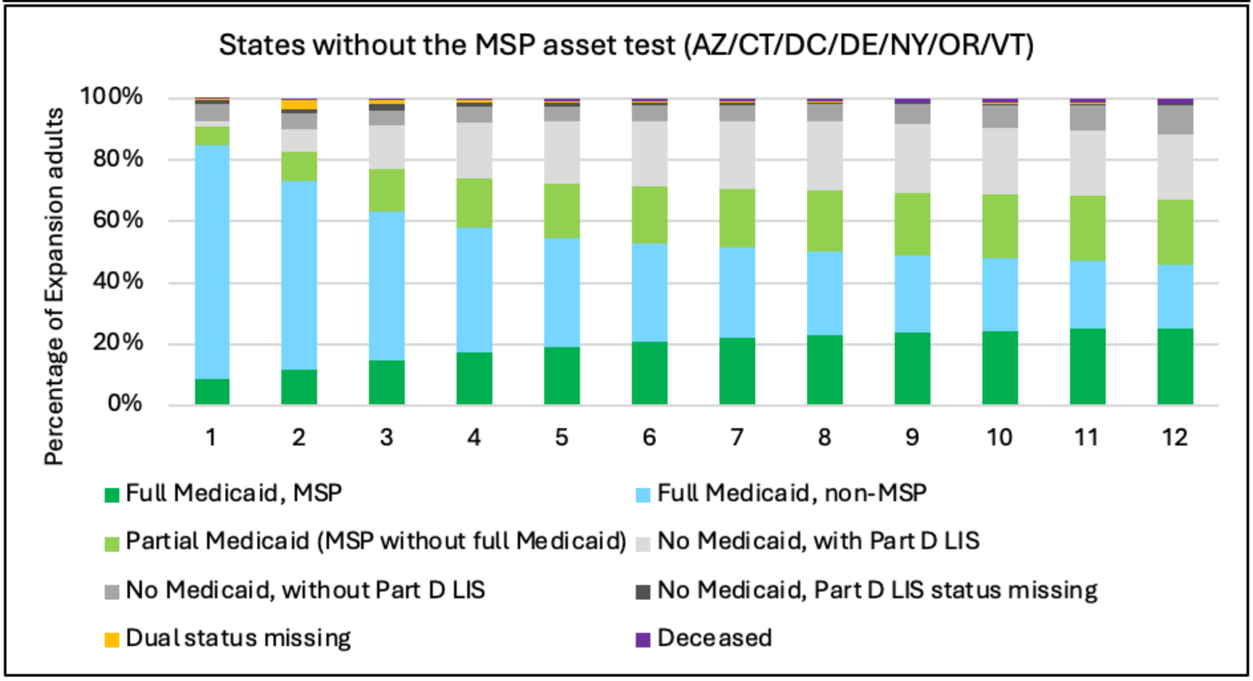

© 2026 Yang Z et al. *JAMA Network Open*

eFigure 4. Medicaid coverage by month over the first Medicare year of expansion adults transitioning to Medicare in 2018, in states with versus without the MSP asset test, by reason for Medicare eligibility

A) Old Age

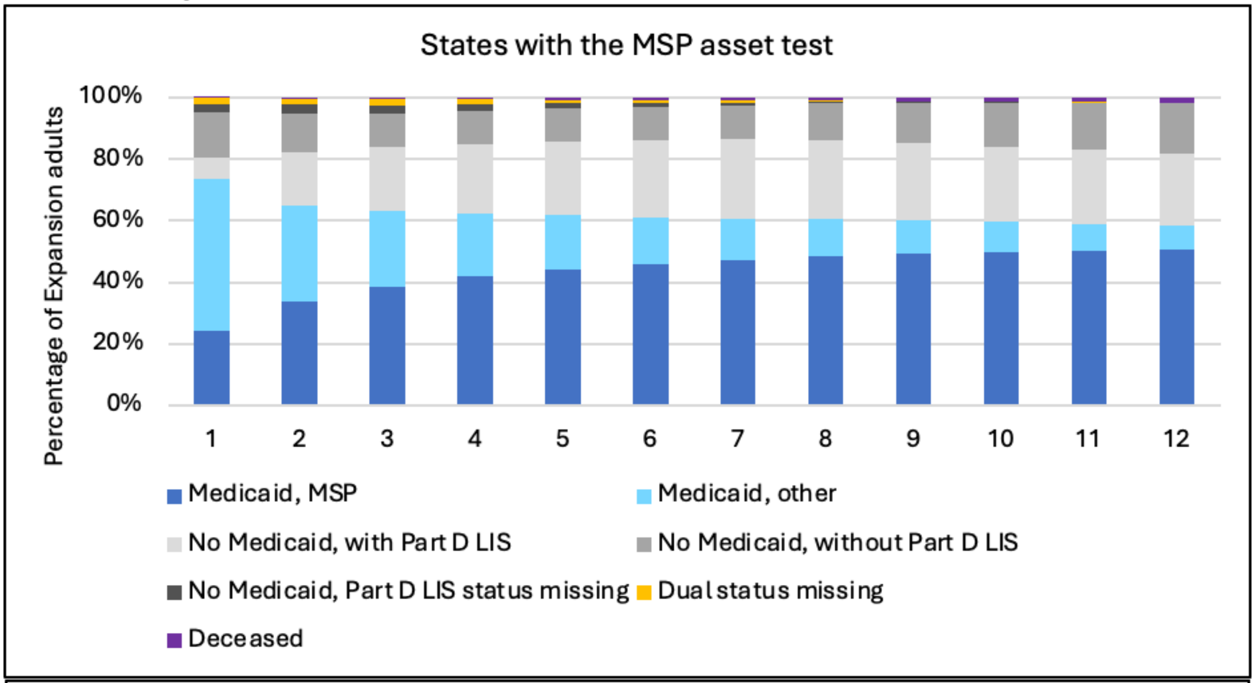

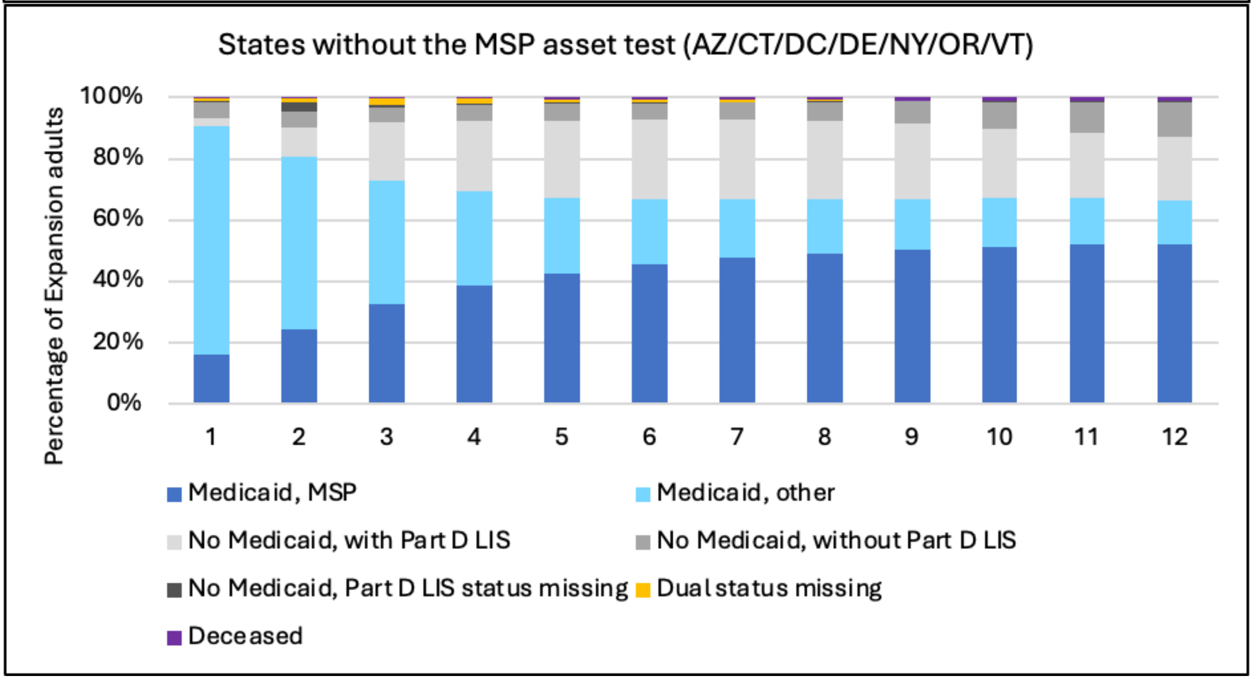

© 2026 Yang Z et al. *JAMA Network Open*

B) Disability

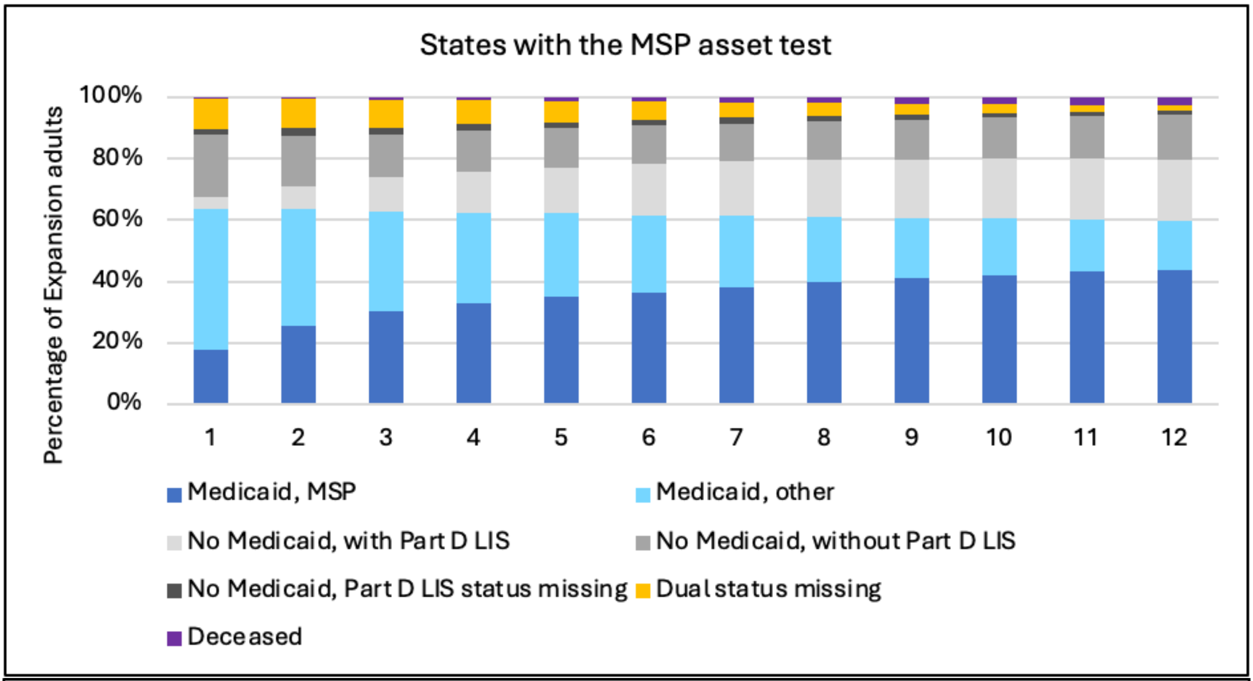

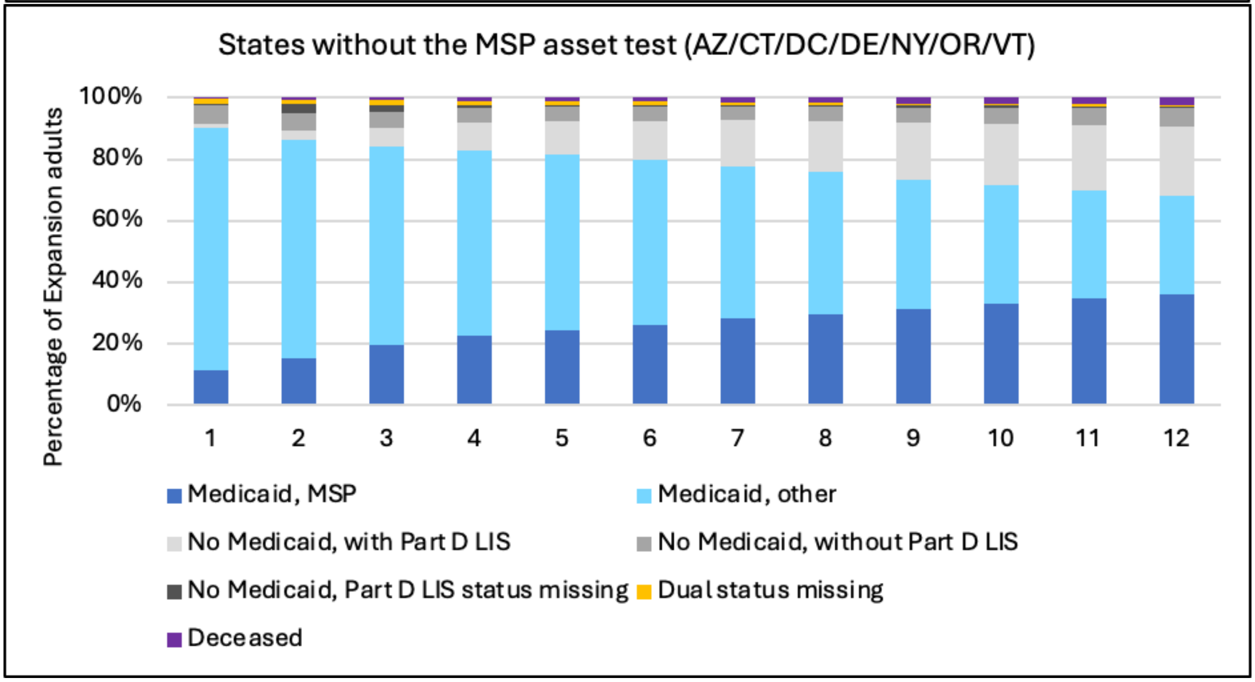

Note: Beneficiaries who qualified for Medicare due to ESRD or both disability and ESRD were not included in this analysis due to their small cell size.

© 2026 Yang Z et al. *JAMA Network Open*

eTable 2. Associations between the MSP asset test and MSP coverage over the first Medicare year of expansion adults transitioning to Medicare in 2018, full results

Outcome	MSP in the 12th month after Medicare			≥6 months of MSP in the first Medicare year			12 months of MSP in the first Medicare year		
	Coef.	95% CI		Coef.	95% CI		Coef.	95% CI	
**Unadjusted for covariates:**									
No MSP asset test	-2%	-18%	15%	-4%	-23%	16%	-4%	-24%	17%
**Adjusted for covariates:**									
No MSP asset test	-1%	-12%	10%	-2%	-17%	12%	-1%	-19%	17%
Original reason for Medicare entitlement (ref.: Old age):									
Disability	-11%	-16%	-5%	-13%	-19%	-8%	-7%	-10%	-3%
ESRD or both disability and ESRD	-16%	-26%	-7%	-20%	-28%	-12%	-15%	-21%	-10%
Female	5%	4%	6%	5%	4%	6%	3%	2%	4%
RTI race and ethnicity (ref.: Non-Hispanic White):									
Asian / Pacific Islander	17%	11%	22%	13%	7%	20%	1%	-5%	8%
Black (or African American)	9%	2%	15%	8%	1%	14%	3%	0%	6%
Hispanic	8%	4%	12%	6%	2%	9%	0%	-3%	3%
CMS-HCC community non-dual score quartile (ref.: Most healthy):									
	4%	3%	5%	4%	2%	5%	1%	1%	2%
	8%	6%	9%	7%	6%	9%	3%	1%	4%
Least healthy	10%	8%	11%	9%	6%	11%	4%	2%	6%
One year before Medicare entry:									
12-month continuous Medicaid enrollment	11%	9%	14%	9%	7%	12%	4%	2%	6%
Any Medicaid comprehensive MCO enrollment	0%	-6%	6%	2%	-4%	7%	1%	-4%	5%
Any Medicaid inpatient utilization	1%	0%	2%	2%	1%	2%	0%	-1%	1%

© 2026 Yang Z et al. *JAMA Network Open*

Any Medicaid LTSS utilization	0%	-3%	3%	-1%	-4%	2%	-3%	-4%	-1%
USDA metro county	-3%	-8%	1%	-5%	-9%	0%	-3%	-6%	-1%
CDC SVI overall ranking quartile (ref.: Least vulnerable):									
	4%	2%	5%	3%	2%	5%	1%	0%	3%
	6%	4%	9%	6%	3%	9%	2%	0%	4%
Most vulnerable	8%	6%	11%	7%	4%	10%	3%	0%	5%
SSI criteria for the ABD pathway (ref.: 209(b); SSI):									
209(b) w/ 100% FPL; SSI w/ 100% FPL	-5%	-24%	14%	-8%	-27%	11%	-2%	-20%	16%
1634	-13%	-26%	0%	-16%	-30%	-3%	-12%	-25%	1%
1634 w/ 100% FPL	-5%	-21%	11%	-8%	-25%	9%	-5%	-24%	15%
Medically needy pathway	-10%	-19%	-1%	-11%	-22%	1%	-7%	-23%	9%
**W/O DQ Atlas unusable states:**									
**Unadjusted for covariates:**									
No MSP asset test	-1%	-18%	16%	-3%	-23%	17%	-3%	-23%	18%
**Adjusted for covariates:**									
No MSP asset test	-1%	-14%	12%	-2%	-19%	15%	0%	-20%	19%

Notes: Both unadjusted and adjusted models excluded beneficiaries with missing dual status or deceased in the relevant month(s) of the outcome (see Figure 1). Adjusted models also excluded beneficiaries with AI/AN, Other, or Unknown race and ethnicity, or missing USDA metro county or CDC SVI quartile data (see Table 1). Standard errors were clustered at the state level.

© 2026 Yang Z et al. *JAMA Network Open*

eTable 3. Associations between the MSP asset test and any Medicaid coverage over the first Medicare year of expansion adults transitioning to Medicare in 2018, full results

Outcome	Medicaid in the 12th month after Medicare			≥6 mos. of Medicaid in the first Medicare year			12 mos. of Medicaid in the first Medicare year		
	Coef.	95% CI		Coef.	95% CI		Coef.	95% CI	
**Unadjusted for covariates:**									
No MSP asset test	8%	-3%	18%	11%	-2%	23%	12%	-3%	28%
**Adjusted for covariates:**									
No MSP asset test	10%	3%	17%	14%	5%	22%	16%	6%	26%
Original reason for Medicare entitlement (ref.: Old age):									
Disability	0%	-3%	3%	2%	-4%	8%	-3%	-9%	2%
ESRD or both disability and ESRD	6%	2%	11%	6%	-1%	14%	-3%	-16%	10%
Female	5%	4%	5%	3%	2%	4%	4%	3%	5%
RTI race and ethnicity (ref.: Non-Hispanic White):									
Asian / Pacific Islander	21%	17%	26%	20%	15%	24%	21%	14%	27%
Black (or African American)	9%	4%	14%	7%	3%	12%	5%	-1%	10%
Hispanic	15%	11%	19%	15%	10%	20%	14%	7%	22%
CMS-HCC community non-dual score quartile (ref.: Most healthy):									
	4%	4%	5%	4%	2%	5%	3%	2%	4%
	8%	7%	9%	7%	5%	9%	6%	5%	8%
Least healthy	11%	9%	13%	10%	7%	12%	9%	7%	11%
One year before Medicare entry:									
12-month continuous Medicaid enrollment	13%	10%	16%	11%	9%	14%	13%	10%	16%
Any Medicaid comprehensive MCO enrollment	0%	-6%	6%	0%	-8%	7%	2%	-7%	11%
Any Medicaid inpatient utilization	1%	0%	2%	1%	0%	2%	0%	-1%	1%

© 2026 Yang Z et al. *JAMA Network Open*

Any Medicaid LTSS utilization	9%	7%	12%	8%	5%	11%	10%	6%	13%
USDA metro county	2%	-2%	6%	3%	-2%	8%	3%	-2%	8%
CDC SVI overall ranking quartile (ref.: Least vulnerable):									
	5%	3%	6%	4%	2%	7%	4%	2%	6%
	9%	5%	12%	8%	5%	12%	8%	4%	12%
Most vulnerable	11%	8%	14%	11%	7%	14%	11%	7%	15%
SSI criteria for the ABD pathway (ref.: 209(b); SSI):									
209(b) w/ 100% FPL; SSI w/ 100% FPL	16%	-4%	35%	17%	-3%	37%	20%	-5%	45%
1634	-2%	-12%	9%	-1%	-14%	12%	0%	-20%	21%
1634 w/ 100% FPL	4%	-8%	17%	6%	-11%	23%	9%	-17%	34%
Medically needy pathway	-10%	-21%	0%	-12%	-27%	3%	-11%	-32%	10%
**W/O DQ Atlas unusable states:**									
**Unadjusted for covariates:**									
No MSP asset test	8%	-4%	21%	11%	-4%	26%	13%	-5%	31%
**Adjusted for covariates:**									
No MSP asset test	8%	0%	17%	11%	1%	21%	13%	1%	25%

Notes: Any Medicaid included MSP (with or without full Medicaid) and full Medicaid without MSP coverage. Both unadjusted and adjusted models excluded beneficiaries with missing dual status or deceased in the relevant month(s) of the outcome (see Figure 1). Adjusted models also excluded beneficiaries with AI/AN, Other, or Unknown race and ethnicity, or missing USDA metro county or CDC SVI quartile data (see Table 1). Standard errors were clustered at the state level.

© 2026 Yang Z et al. *JAMA Network Open*

eTable 4. Associations between the MSP asset test and MSP coverage only without full Medicaid over the first Medicare year of expansion adults transitioning to Medicare in 2018

Outcome	MSP without full Medicaid in the 12th month after Medicare			≥6 months of MSP without full Medicaid in the first Medicare year			12 months of MSP without full Medicaid in the first Medicare year		
	Coef.	95% CI		Coef.	95% CI		Coef.	95% CI	
**Unadjusted for covariates:**									
No MSP asset test	3%	-14%	20%	0%	-16%	16%	0%	-9%	9%
**Adjusted for covariates:**									
No MSP asset test	-5%	-15%	5%	-6%	-18%	6%	0%	-8%	8%

Notes: Both unadjusted and adjusted models excluded beneficiaries with missing dual status or deceased in the relevant month(s) of the outcome (see Figure 1). Adjusted models also excluded beneficiaries with AI/AN, Other, or Unknown race and ethnicity, or missing USDA metro county or CDC SVI quartile data (see Table 1). Standard errors were clustered at the state level.

© 2026 Yang Z et al. *JAMA Network Open*

eTable 5. Associations between the MSP asset test and MSP or any Medicaid coverage over the first Medicare year of expansion adults transitioning to Medicare in 2018, by reason for Medicare eligibility

A) Old Age

Outcome	MSP in the 12th month after Medicare			≥6 months of MSP in the first Medicare year			12 months of MSP in the first Medicare year		
	Coef.	95% CI		Coef.	95% CI		Coef.	95% CI	
**Unadjusted for covariates:**									
No MSP asset test	1%	-15%	18%	0%	-20%	20%	-4%	-28%	20%
**Adjusted for covariates:**									
No MSP asset test	5%	-4%	14%	5%	-7%	17%	1%	-18%	20%

Outcome	Medicaid in the 12th month after Medicare			≥6 mos. of Medicaid in the first Medicare year			12 mos. of Medicaid in the first Medicare year		
	Coef.	95% CI		Coef.	95% CI		Coef.	95% CI	
**Unadjusted for covariates:**									
No MSP asset test	8%	-4%	20%	7%	-8%	22%	9%	-7%	26%
**Adjusted for covariates:**									
No MSP asset test	11%	5%	18%	12%	4%	20%	15%	5%	24%

B) Disability

C) Outcome	MSP in the 12th month after Medicare			≥6 months of MSP in the first Medicare year			12 months of MSP in the first Medicare year		
	Coef.	95% CI		Coef.	95% CI		Coef.	95% CI	
**Unadjusted for covariates:**									
No MSP asset test	-9%	-29%	11%	-13%	-34%	8%	-3%	-17%	11%
**Adjusted for covariates:**									
No MSP asset test	-11%	-24%	2%	-14%	-31%	2%	-3%	-18%	11%

© 2026 Yang Z et al. *JAMA Network Open*

Outcome	Medicaid in the 12th month after Medicare			≥6 mos. of Medicaid in the first Medicare year			12 mos. of Medicaid in the first Medicare year		
	Coef.	95% CI		Coef.	95% CI		Coef.	95% CI	
**Unadjusted for covariates:**									
No MSP asset test	8%	-2%	18%	19%	7%	31%	17%	2%	32%
**Adjusted for covariates:**									
No MSP asset test	9%	1%	16%	19%	9%	30%	19%	7%	31%

Notes: Beneficiaries who qualified for Medicare due to ESRD or both disability and ESRD were not included in this analysis due to their small cell size. Any Medicaid included MSP (with or without full Medicaid) and full Medicaid without MSP coverage. Both unadjusted and adjusted models excluded beneficiaries with missing dual status or deceased in the relevant month(s) of the outcome (see Figure 1). Adjusted models also excluded beneficiaries with AI/AN, Other, or Unknown race and ethnicity, or missing USDA metro county or CDC SVI quartile data (see Table 1). Standard errors were clustered at the state level.

© 2026 Yang Z et al. *JAMA Network Open*
