# Supplementary material for: Medicaid Retention After Transition to Medicare Among Adults With Expansion Coverage
Source: JAMA Netw Open. 2026 Apr 22;9(4):e268560. doi: 10.1001/jamanetworkopen.2026.8560 (PMC13103809; doi:10.1001/jamanetworkopen.2026.8560)
Supplement: Supplement 2. — Data Sharing Statement [file jamanetwopen-e268560-s002.pdf]

Data Sharing Statement

Yang. Medicaid Retention After Transition to Medicare Among Adults With Expansion Coverage. *JAMA Netw Open*. Published April 22, 2026.
doi:10.1001/jamanetworkopen.2026.8560

Data
Data available: No

Additional Information
Explanation for why data not available: The data cannot be made available to others due to the terms and conditions established by our data use agreement with the Centers for Medicare & Medicaid Services (CMS).
